# PDK1 inhibitor GSK2334470 exerts antitumor activity in multiple myeloma and forms a novel multitargeted combination with dual mTORC1/C2 inhibitor PP242

**DOI:** 10.18632/oncotarget.16642

**Published:** 2017-03-29

**Authors:** Chunmei Yang, Xianbo Huang, Hui Liu, Feng Xiao, Jueying Wei, Liangshun You, Wenbin Qian

**Affiliations:** ^1^ Institute of Hematology, the First Affiliated Hospital, College of Medicine, Zhejiang University, Hangzhou, 310003, P.R. China

**Keywords:** multiple myeloma, PDK1, mTOR, PTEN

## Abstract

A deeper understanding of the complex pathogenesis of multiple myeloma (MM) continues to lead to novel therapeutic approaches. Prior studies suggest that 3-phosphoinositide-dependent kinase 1 (PDK1) is expressed and active, acting as a crucial regulator of molecules that are essential for myelomagenesis. In the present study, we show that GSK2334470 (GSK-470), a novel and highly specific inhibitor of PDK1, induces potent cytotoxicity in MM cell lines including Dexamethasone-resistant cell line, but not in human normal cells. Insulin-like growth factor-1 could not rescue GSK-470-induced cell death. Moreover, GSK-470 down-modulates phosphor-PDK1, thereby inhibiting downstream phosphor-AKT at Thr308 and mTOR complex 1 (mTORC1) activity. However, GSK-470 could not affect mTORC2 activity and phosphor-AKT at Ser473. RPMI 8226 and OPM-2 cells with low expression of PTEN show relative resistant to GSK-470. Knockout of PTEN by shRNA resulted in a partial reversion of GSK-470-mediated growth inhibition, whereas overexpression of PTEN enhanced myeloma cell sensitivity to GSK-470, suggesting that the sensitivity to GSK-470 is correlated with PTEN expression statue in MM cells. Combining PP242, a dual mTORC1/C2 inhibitor, with GSK-470, had greater antimyeloma activity than either one alone *in vitro* and in MM xenograft model established in immunodeficient mice. In particular, this combination was able to result in a complete inhibition of mTORC1/C2 and full activity of AKT. Together, these findings raise the possibility that combining PDK1 antagonist GSK-470 with mTORC1/C2 inhibitors may represent a novel strategy against MM including drug-resistant myeloma, regardless of PTEN expression status.

## INTRODUCTION

Multiple myeloma (MM) is a molecularly/symptomatically heterogeneous B-cell malignancy characterized by the accumulation of clonal malignant plasma in bone marrow [1]. Over the past decade, patients with MM are living longer and better due to the introduction of several classes of drugs recently approved in the treatment of MM, which include the immunomodulators (thalidomide, lenalidomide, and pomalidomide), proteasome inhibitors (bortezomib, carfilzomib, and ixazomib), and the histone deacetylase (HDAC) blocker panobinostat [1–3]. Supplementation of MM therapy with autologous stem cell transplantation has further improved median overall survival of the patients, especially in the patients with standard risk [4]. More recently, the U.S. Food and Drug Administration (FDA) approved for use in MM two monoclonal antibodies, daratumumab and elotuzumab, both directed against glycoproteins expressed on the surface of MM cells [5, 6]. Despite these significant improvements, the cure of patients with MM remains challenging and difficult.

In MM, aberrant activation of several signal transduction pathways results in cellular proliferation and drug-resistance of tumor cells [1]. Therefore, these signaling cascades may be attractive targets for the development of innovative therapeutic strategies for MM patients. The PI3K/AKT/mTOR signaling pathway is frequently activated in myeloma. Loss of the tumor suppressor PTEN, mutations in receptor tyrosine kinases, as well as activating PI3K mutations all result in elevated levels of phosphatidylinositol phosphates, including PIP3 that activates the AKT pathway [7–10]. PIP3 recruits phosphoinositide-dependent protein kinase 1 (PDK1) and AKT to the plasma membrane wherein PDK1 directly phosphorylates Thr308 residues of AKT [11], but requires mTOR complex 2 (mTORC2)-induced AKT phosphorylation on Ser473 to confer its full activation [12]. Currently, AKT is considered to be the main effector of PDK1 in cancer, thereby targeting PDK1 may provide an opportunity for developing novel therapeutics for cancer. Indeed, it has been recently shown that GSK2334470 (GSK-470), a novel and highly specific inhibitor of PDK1 [13], inhibits growth, induces cell cycle arrest and overcomes drug resistance in human cancer cells [14–16]. In MM, it was demonstrated that PDK1 is expressed and active in all eleven MM-derived cell lines, regardless of the type of cytogenetic abnormality or the status of upstream signaling molecules, and that genetic or pharmacological (BX-912) inhibition of PDK1 caused the growth inhibition and the induction of apoptosis, and augmented the *in vitro* cytotoxic effects of antimyeloma agents such as melphalan, etoposide, or bortezomib [17].

Recently, a number of small molecular inhibitors of PDK1, such as UCN-01, dibenzo [c,f]- [2, 7] naphthyridine derivatives, celecoxib derivatives, BX-795 and BX-912, have been described that are poorly specific and/or ineffective at suppressing PDK1-dependent pathway *in vivo* [18, 19]. Whereas, GSK-470 has been shown to effectively inhibit PDK1 at very low concentrations, but do not suppress the activity of 93 other protein kinases including 13 AGC family of protein kinases [13], suggesting it is a highly specific and potent inhibitor of PDK1. Nevertheless, its effect and the mechanism of action in the MM context need to be studied. In the present study, we addressed the molecular mechanisms of the anti-MM action of GSK-470 and showed that GSK-470 inhibits cellular proliferation and induces apoptosis. However, myeloma cell lines with absence or dysfunction of PTEN are relatively resistant to the drug-induced cell death. Therefore, we next evaluated to which extent dual targeting of the PDK1 and mTORC1/C2 pathways can enhance the antimyeloma efficacy. The findings of the present study provide a rationale for combination therapy using GSK-470 and PP242, a mTORC1/C2 inhibitor, for the treatment of MM.

## RESULTS

### GSK-470 inhibits cellular proliferation and induces apoptosis possibly related to the function of PTEN in MM cell lines

**Figure 1:**
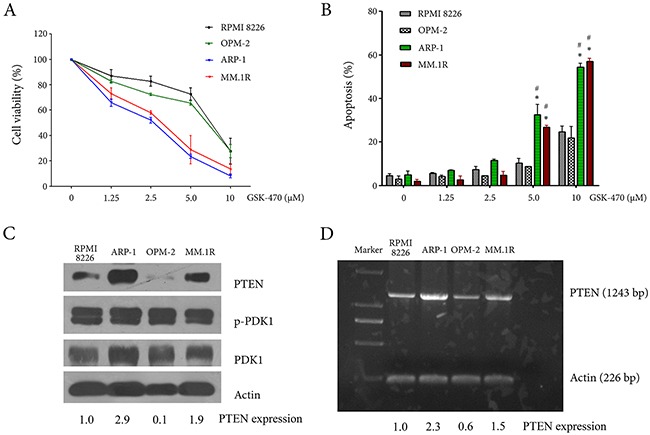
Anti-myeloma effect of GSK-470 and the constitutive expression of PTEN and PDK1 in myeloma cell lines **(A)**. RPMI 8226, OPM-2, ARP-1 and MM.1R cell lines were plated in 96-well plates and treated with GSK-470 at indicated concentrations for 24 h. The MTT assay was then used to quantify the viability of cells. Data are presented as the mean ± SD of three independent experiments. **(B)**. Four kinds of MM cell lines were harvested at 24 h after treatment with different concentrations of GSK-470. Apoptosis was analyzed by flow cytometry after dual staining of cells with annexin V and propidium iodide (PI). The percentage of gated cells that were apoptotic (annexin V single positive and annexin V/PI double positive) was assessed. The results are shown as the average of three independent experiments; bars, ± SD. * *P* < 0.05 *vs*. RPMI 8226; ^#^*P* < 0.05 *vs*. OPM-2. **(C)** and **(D)**. Baseline expression of PTEN and PDK1 in MM cell lines was assessed by Western blotting analysis and RT-PCR analysis, respectively. The difference in the level of PTEN expression was semi-quantitatively detected by densitometry and expressed as a ratio.

The effect of GSK-470 on growth of MM cell lines was determined by an MTT assay. A dose-dependent growth inhibition was observed in all tested MM cell lines following the treatment of GSK-470. The results showed that ARP-1 and MM.1R cells were sensitive to GSK-470 with IC50 values of 3.98 μM and 4.89 μM, respectively. Whereas, RPMI 8226 and OPM-2 cells were relatively resistant to GSK-470 with IC50 values of 8.4 μM and 10.56 μM, respectively (Figure 1A). To assess the mechanism of toxicity, the cell lines treated with GSK-470 at the indicated concentrations were analyzed for expression of Annexin V by FACS analysis concomitantly with PI staining. In accordance with the data on MTT assay, ARP-1 and MM.1R cells showed higher rates of apoptosis than RPMI 8226 and OPM-2 cells (Figure 1B). We next assessed mRNA and protein expression of PTEN and PDK1, respectively, in MM cell lines because PDK1 inhibition had been shown to be fail to prevent tumor growth in PTEN-deficient animal models [20]. As shown in Figure 1C and 1D, there is no significant difference in the level of PDK1 and phospho-PDK1; however mRNA and protein expressions of PTEN in ARP-1 and MM.1R cells were higher than that in RPMI 8226 and OPM-2 cells that had been demonstrated to be loss of PTEN due to the deletion spacing from exon 3 to 7 [7, 21]. Correctively, our data suggested that GSK-470 inhibited proliferation and induced apoptosis of MM cells, and anti-myeloma effect of GSK-470 might correlate with the level of PTEN expression.

### GSK-470 induces apoptosis by inhibiting the phosphorylation of PDK1 and its downstream AKT/mTOR pathway

**Figure 2:**
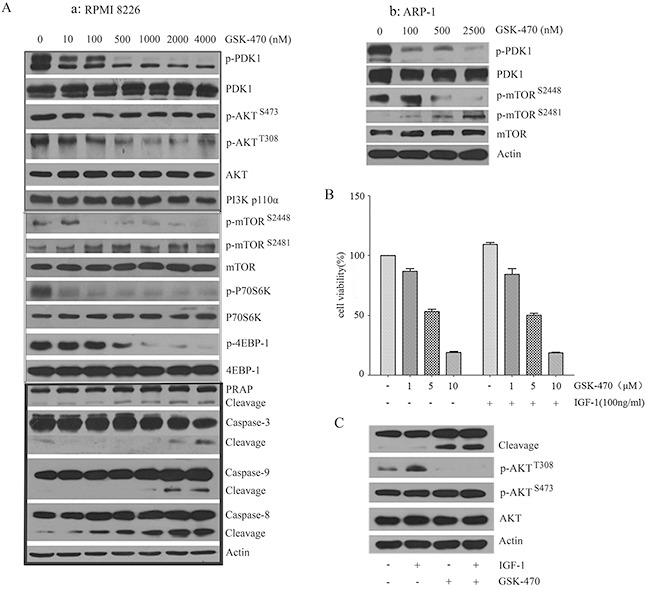
GSK-470 induces apoptosis and overcomes the protective effect of IGF-1 via inhibiting PDK1/AKT/mTORC1 pathway **(A)**. RPMI 8226 and ARP-1 cells treated with GSK-470 at the indicated concentrations for 24 h were taken for the detection of activation of caspase pathway and PDK1/AKT/mTORC1 pathway using western blotting analysis. **(B)** and **(C)**. For stimulation with recombinant IGF-1, RPMI 8226 cells were serum starved for 2 h, followed by incubation with or without 100 ng/ml IGF-1 for 2 h. Then co-cultured with indicated concentrations of GSK-470 for 24 h. Cell growth was assessed by an MTT assay. PARP activation and phosphorylation of AKT were checked by Western blotting analysis. Actin was used as a loading control.

To identify the potential cellular target of GSK-470 and clarify the underlying molecular mechanism in GSK-470-induced cell apoptosis, we first examined the effects of GSK-470 on PDK1 and its downstream AKT expression by Western blot analysis (Figure 2A), GSK-470 dose-dependently decreased phosphorylation of PDK1 at Ser241 and AKT at Thr308 in RPMI 8226 and ARP-1 cells. As expected, GSK-470 also strong inhibited phosphorylation of mTOR on Ser2448, a marker for mTORC1 activity, as well as phosphorylation of 4E-BP1 and p70S6K, the best characterized targets of mTORC1. However, level of phospho-mTOR at Ser2481 (a marker for the presence of mTORC2 complex) and its downstream phospho-AKT (Ser473) were not significantly affected. Consistent with the inhibition of the PDK1 and AKT/mTOR activity, same doses of the drug dose-dependently activated caspase pathway, as evidenced by cleavage and activation of caspase-9, -8, -3 and downstream PRAP (Figure 2A), which indicates the activation of apoptosis.

Insulin-like growth factor-1 (IGF-1) and its receptor (IGF-1R) play an important role in MM pathogenesis. IGF-1/IGF-1R triggers a variety of signaling cascades including AKT, mediating proliferation, survival, and drug resistance of MM cells [22, 23]. To investigate whether GSK-470-induced inhibition of phospho-AKT can diminish the stimulatory effect of IGF-1, anti-proliferation effect of GSK-470 on RPMI 8226 cells in the presence of exogenous IGF-1 was evaluated. IGF-1 could not protect against GSK-470-induced growth inhibition (Figure 2B). Western blotting analysis showed that IGF-1 treatment resulted in activation of AKT, as evidenced by increased level of phosphorylated AKT at Thr308. However, it fail to reverse suppression of phosphor-AKT at Thr308 and induction of apoptosis induced by GSK-470 (Figure 2C).

### Relationship between anti-myeloma efficacy of GSK-470 and PTEN expression

**Figure 3:**
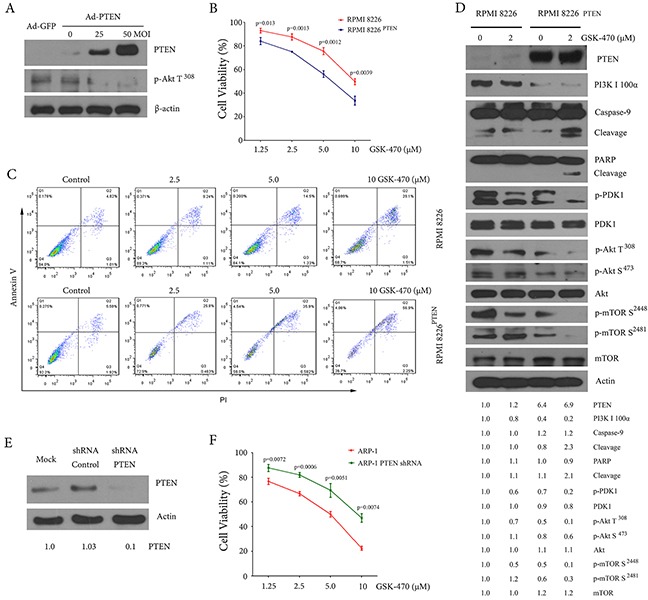
PTEN status affects GSK-470-mediated anti-myeloma effect **(A)**. After infection of RPMI 8226 cells for 48 h with the adenovirus vectors carrying PTEN gene (Ad-PTEN) or GFP gene (Ad-GFP), respectively, whole cell lysates were analyzed for PTEN protein expression and phosphorylation of AKT (Thr308) using Western blotting analysis. **(B)** and **(C)**. RPMI 8226 and RPMI 8226^PTEN^ cells were treated with the indicated concentrations of GSK-470 for 24 h. Cellular proliferation was assessed by an MTT assay. Data are presented as the means ± SD of three independent experiments. Apoptosis was evaluated by annexin V/PI staining, and representative results of three samples are shown. **(D)**. RPMI 8226 and RPMI 8226^PTEN^ cells were treated with or without 2 μM GSK-470 for 24 h. Whole-cell lysates were subjected to Western blotting to assess active PDK1 and activation of PI3K/ AKT /mTOR pathway. The cleavage of PARP and caspase-9 also was assessed. The difference in the level of protein expression was semi-quantitatively determined by densitometry and expressed as a ratio. Actin was used as internal standard. **(E)**. ARP-1 cells were transfected with adenovirus vectors containing PTEN shRNA or control shRNA. Cell lysates were prepared for detection of PTEN protein expression by Western blotting. PTEN expression was semi-quantitatively detected by densitometry. Actin served as a loading control. **(F)**. ARP-1 and ARP-1^PTEN ShRNA^ cells were treated with indicated concentrations of GSK-470 for 24 h. Cell viability was assessed by an MTT assay. Data are presented as the means ± SD of three independent experiments.

To investigate whether overexpression of PTEN enhances sensitivity of GSK-470 in MM cells, we infected RPMI 8226 cells that have low expression of PTEN with a PTEN adenoviral expression vector, and analyzed cellular proliferation inhibition and induction of apoptosis by GSK-470. As shown in Figure 3A, overexpression of PTEN resulted in down-regulation of phosphor-AKT (Thr308). Furthermore, RPMI 8226 cells infected with Ad-PTEN (RPMI 8226^PTEN^) are more sensitive to GSK-470-induced cell death than parent RPMI 8226 cells (Figure 3B, 3C and 3D). To further evaluate if PTEN was related to sensitivity of MM cells to GSK-470, we examined the effect of knockout PTEN by shRNA on cell death induced by GSK-470 in ARP-1 cells. We chosen ARP-1 cells that was infected with an adenoviral vector expressing PTEN shRNA (Figure 3E), because high expression of PTEN was observed in ARP-1 cells. As expected, knockout of PTEN resulted in a partial reversion of cellular growth inhibition (Figure 3F).

PTEN is involved in the regulation of the PI3K/AKT/mTOR pathway through its lipid phosphatase activity [24, 25]. A loss of PTEN functions results in AKT phosphorylation [26]. We thus examined whether overexpression of PTEN affects PI3K/AKT/mTOR pathway in RPMI 8226 cells and found that PTEN overexpression led to significant decreases in basal level of phosphor-PDK1 (Ser241), phosphor-AKT (Ser473 and Thr308) and phosphor-mTOR (Ser2448 and Ser2481). In addition, PTEN overexpression also inhibited the expression of p110, a class I PI3K isoform. These effects were enhanced by the treatment of GSK-470 (Figure 3D).

### Synergistic activity of GSK-470 with mTOR inhibitor PP242

**Figure 4:**
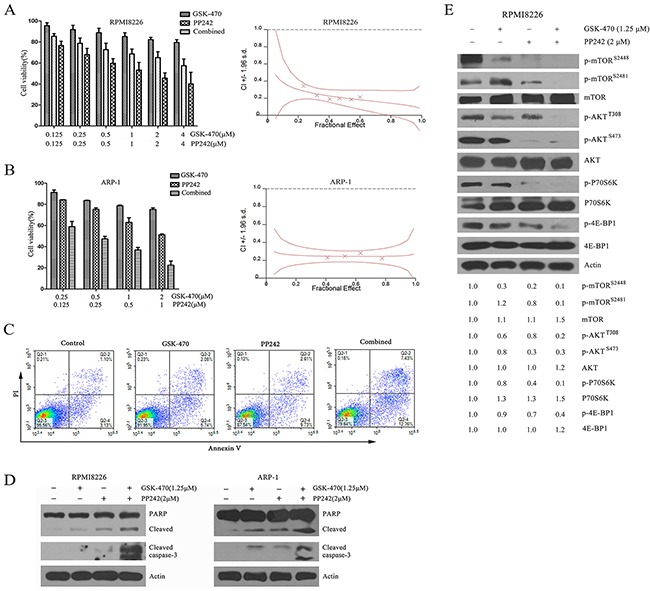
Combination of GSK-470 with PP242 enhances cytotoxicity against MM cells via completely inhibited the phosphorylation of AKT and activity of mTORC1/C2 **(A)** and **(B)**. RPMI 8226 and ARP-1cells were treated with a series of doses of GSK-470 or/and PP242 for 24 h, and cell viability was determined by an MTT assay. Data are presented as the means ± SD of three independent experiments. The combination index was calculated by Calcusyn software. **(C)**. RPMI 8226 cells were treated with GSK-470 (1.25 μM), PP242 (2 μM) or in combination for 24 h. Apoptosis was analyzed by flow cytometry after dual staining of cells with annexin V and propidium iodide (PI). **(D)**. RPMI 8226 and ARP-1 cells were treated with GSK-470 (1.25 μM) alone or in combination with PP242 (2 μM) for 24 h. Cleavage of PARP and caspase-3 were analyzed by western blotting analysis. Actin was used as a loading control. **(E)**. RPMI 8226 cells were treated with GSK-470 (1.25 μM), PP242 (2 μM) or in combination for 24h. The expression and phosphorylation of AKT, mTOR and its downstream targets were also determined. Actin was used as protein loading control. The difference in the level of protein expression was semi-quantitatively determined by densitometry and expressed as a ratio.

Given the facts that GSK-470 failed to inhibit phospho-mTOR at Ser2448 and its downstream phospho-AKT (Ser473), which was demonstrated in this study, and that dual mTORC1/C2 inhibitors is much more active than mTORC1 inhibition alone in myeloma cells [27], we examined whether PP242, a non-rapalog agent that targets simultaneously mTORC1 and mTORC2 [28], enhances GSK-470-mediated cell death. For this, we treated RPMI 8226 and ARP-1 cells with a series of doses of GSK-470 or/and PP242. As revealed by MTT assay, GSK-470 (2 μM) or PP242 (2 μM) decreased the cell viability of RPMI 8226 cells to 82.11% or 65.08%, respectively, whereas the viability of RPMI 8226 cells treated with the combination therapy decreased to 45.5% (Figure 4A). Isobologram analyses confirmed a synergistic anti-myeloma activity of GSK-470 with PP242 (combination index [CI] <0.4). Similar result was observed in ARP-1 cells that is sensitive to GSK-470 (Figure 4B) and in human primary myeloma cells (Supplementary Figure 2A). PP242 also potentiated the GSK-470-induced apoptosis as judged by FACS analysis (Figure 4C), cleavage and activation of caspase-8, -9 and downstream molecules caspase-3 and PRAP (Figure 4D). To analyze the mechanism whereby this combination treatment mediates synergistic anti-myeloma toxicity, we next used immunoblotting to examined the effect of GSK-470, PP242, or combination on AKT/mTOR pathway. A previous study has shown that PP242 could inhibit mTORC1 and mTORC2 activity in MM cells [29]. Consistent with this report, PP242 inhibited not only the level of phosphorylated mTOR (Ser2448) as well as phosphorylation of the mTORC1 substrates p70S6kinase and 4E-BP-1, but also phosphorylation of mTOR (Ser2481) and AKT on Ser473, a mTORC2 substrate (Figure 4E and Supplementary Figure 2B). Importantly, combining GSK-470 and PP242 led to complete elimination of phosphorylated AKT (Ser473/Thr308) and activity of mTORC1 as well as mTORC2, although GSK-470 alone failed to prevent activation of mTORC2 (Figure 4E).

### *In vivo* activity of GSK-470/PP242 combined treatment in a MM xenograft model

**Figure 5:**
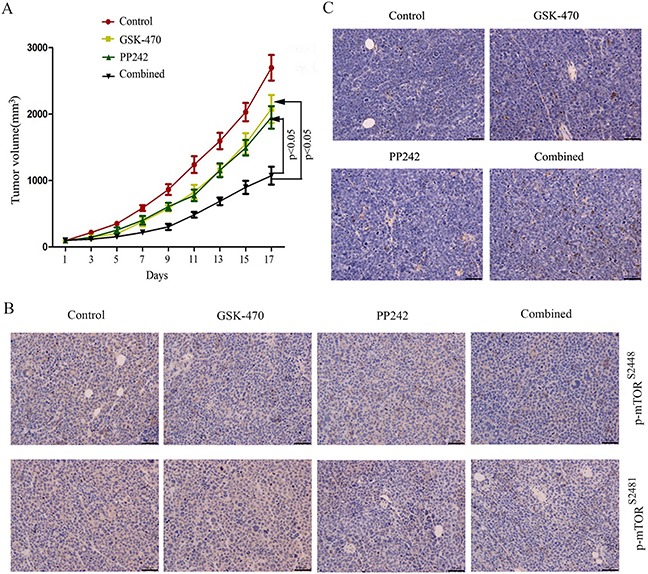
Antitumoral efficacy of GSK-470 combined with PP242 *in vivo* **(A)**. RPMI 8226 xenografts were established in mice (n=8) and treated with GSK-470, PP242 or in combination. Tumor growth was measured at the indicated times. Data are presented as the tumor mean volume ± SD. **(B)**. Tumor sections were excised and analyzed for induction of apoptosis by TUNEL staining. Original magnification, ×40. **(C)**. Protein expression of p-mTOR (Ser2448/Ser2481) in tumors on day 5 after treatment by immunohistochemical staining.

To assess whether the synergistic antimyeloma effects demonstrated *in vitro* could be confirmed *in vivo*, we used a RPMI 8226 xenograft flank model because RPMI 8226 is relatively resistant to not only GSK-470 (Figure 1A) but also PP242 (29). As shown in Figure 5A, tumors in untreated mice grew rapidly and reached the end point size (an average tumor volume of 2696.02 ± 193.30 mm^3^) at 17 days. GSK-470 or PP242 treatment produced a modest tumor volume reduction compared with untreated mice. However, combination treatment with GSK-470 and PP242 proved very efficacious as shown by significant inhibition of tumor growth compared with GSK-470 (*P*<0.05) or PP242 alone (*P*<0.05).

Next, immunohistochemical analyses of the xenograft tumors that were extracted at 5 days after treatment were performed to determine if the observed *in vivo* effects were mediated by mTORC1/C2 inhibition. Decreased phosphor-mTOR (Ser2448) expression was observed in GSK-470 treated tumor sample, whereas PP242 inhibited phosphorylation of mTOR at both Ser2448 and Ser2481. This inhibition was enhanced by GSK-470 and PP242 co-treatment (Figure 5B). Notably, a significant increase of apoptosis was also demonstrated by TUNEL assay in the GSK-470/PP242 group compared with control or single-agent groups (Figure 5C).

## DISCUSSION

PDK1 plays a pivotal role in modulating PI3K-pathway signaling, and is involved in the regulation of cell metabolism, proliferation, and survival of tumor cells [30, 31]. Recently, several lines of evidence indicate that PDK1 can be considered as a promising target for anticancer drugs and various classes of small molecular inhibitor targeting PDK1 have been proposed [32–34]. In MM, PDK1 is generally active and higher expression than other hematopoietic lineages [17, 35, 36]. Activation of PDK1 is essential for myelomagenesis by regulating RSK2, AKT, c-MYC, IRF4 or cyclin Ds, which accelerates the drug resistance and the disease progression [17]. In this study, we first showed that GSK-470, a highly specific inhibitor of PDK1, directly inhibits growth of MM cell lines, including Dexamethasone-resistant cell line. The IC50 values for the 4 myeloma cell lines were 3.98-10.56 μM. In contrast, human normal L02 cells and human umbilical vein endothelial cells (HUVEC) were much less sensitive to GSK-470 (Supplementary Figure 1). This observation is consistent with a previous study showing similar effects of PDK1 inhibitors BX-912 and AR-12 in myeloma cells, with IC50 ranging from 2.5 to 12.8 μM [17]. Importantly, our analysis also revealed that the level of sensitivity of MM cell lines to GSK-470 was affected by PTEN expression, and that PTEN knockdown resulted in drug resistant while restoration of PTEN expression led to increased cell death in response to GSK-470 treatment. The data support the notion that inhibition of PDK1 is not sufficient to prevent tumor formation and progression resulting from loss of PTEN [20].

Mechanistically, although GSK-470 significantly inhibited phosphorylation of PDK1 (Ser241) and mTOR on Ser2448, a marker for mTORC1 activity, it could not completely suppress phosphor-AKT (Ser473) in RPMI 8226 cells with low expression of PTEN, even at the higher concentration of 4 μM (Figure 2A). This is also consistent with the finding that in epithelial cells, combined pharmacological and genetic inactivation of PDK1 does not suppress AKT activation in a PTEN-deficient setting [20]. PTEN, a tumor suppressor gene, negatively regulates the PI3K/AKT/mTOR pathway through its lipid phosphatase activity [24]. More recent studies have shown that PTEN also negatively regulates mTORC2 formation [37, 38]. We show here that PDK1 inhibition by GSK-470 does not lead to a change in the phosphorylation level of mTOR Ser2481. However, the overexpression of PTEN in RPMI 8226 cells resulted in significant decreased level of phosphor-AKT (Ser473 and Thr308) and inhibition of mTORC1/C2 activity as evidenced by dephosphorylation of mTOR on Ser2448 and Ser2481. Moreover, this inhibitory effect was enhanced after treatment with GSK-470. Taken together, our results provide a novel insight on the molecular mechanism of PDK1 inhibitor resistance in MM.

**Figure 6:**
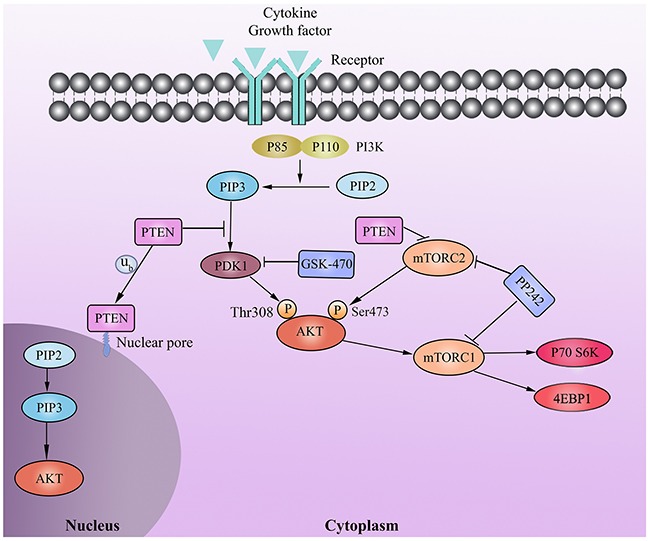
Schematic representation of the signal pathways involved in the combination therapy of GSK-470 and PP242 in MM PDK1 inhibitor GSK-470 inhibits the phosphorylation of AKT at Thr308 and mTOR at Ser2448, as well as its direct targets (4E-BP1 and p70S6K). PTEN affects the antimyeloma effects of GSK-470 by regulating not only PDK1 activity but also mTORC2 formation, thereby inhibiting phosphor- AKT on Ser473. Combined with GSK-470 and mTORC1/C2 inhibitor PP242, can completely inhibit phosphorylated AKT (Ser473/Thr308) and activity of mTORC1 as well as mTORC2, regardless of the PTEN expression status.

Loss or inactivation of PTEN via somatic mutation or epigenetic silencing is a frequent event in many cancers [39]. Although PTEN plays a crucial role in regulating hematopoietic cell proliferation, cell death, and malignant transformation, its mutations are uncommon in MM patients [40]. However, it was reported that the level of PTEN protein expression was significantly lower in patients with advanced myeloma than in controls, indicating the relationship between abnormal expression of PTEN and disease progression [41]. Multiple mechanisms seem to be implicated in PTEN inactivation in MM cells including epigenetic silencing by gene promoter methylation [42] and post-translational modifications [43–45], thus epigenetic silencing may underlie low expression of PTEN in RPMI 8226 cells. Increasing lines of evidence indicate that targeting the mTOR pathway may represent an efficient strategy against MM [1–3, 29]. *In vitro* and *in vivo* studies showed that dual mTORC1/C2 inhibitor is more active against myeloma cells than mTORC1 inhibition alone (rapamycin) that could results in the feedback activation of AKT [27, 46]. But one major weakness of mTORC1/C2 inhibitor treatment is that it induces upregulation of IGF-1 receptor phosphorylation in MM cell lines, which rescues myeloma cells from apoptosis despite mTOR kinase inhibition and mTORC2/AKT blockage [47]. Given these observation and the fact that exogenous IGF-1 did not reverse GSK-470-induce cell death and GSK-470 could not inhibit mTORC2, there is a need to combine dual mTORC1/C2 inhibitor with GSK-470 to target and overcome these resistance mechanisms. In this study, we show that GSK-470 triggers significant synergistic cytotoxicity by apoptosis of both the GSK-470-sensitive and -resistant MM cells *in vitro* and *in vivo* when used in combination with a mTORC1/C2 inhibitor PP242 [1, 48]. Importantly, this combination treatment resulted in a complete inhibition of phosphorylation of AKT on Thr308 and downstream mTORC1 as evidenced by diminished phosphor-4E-BP1 and phosphor-P70S6K. Furthermore, cotreatment with low concentration of GSK-470 (1.25 μM) and PP242 (2 μM) potently inhibited phosphorylation of mTOR at Ser2481 and its direct target AKT at Ser473 (Figure 6). Together, our data provide the rationale for a novel treatment strategy combining selective PDK1 inhibitor and mTORC1/C2 inhibitor to improve MM patient outcome, regardless of the PTEN status.

In summary, PDK1 inhibitor GSK-470 in combination with dual mTORC1/C2 inhibitor PP242 shows significant synergistic antimyeloma effects regardless of the PTEN status in MM cells, providing the framework for clinical trials of combined therapy to improve patient outcome in MM.

## MATERIALS AND METHODS

### Cell culture and reagents

Human MM cell line RPMI 8226 and HUVEC were purchased from American Type Culture Collection (Rockville, MD, USA). Human normal hepatocytes L02 cell line was obtained from the Shanghai Cell Collection (Shanghai, China). OPM-2 and ARP-1 were provided by Prof. Cai Z (Zhejiang University, Hangzhou, China), and Dexamethasone-resistant MM cell line (MM.1R) was kindly provided by Steven Rosen (Northwestern University, Chicago, IL, USA). MM cell lines were cultured in RPMI1640 (Hyclone Laboratories, Logan, UT, USA), HUVEC and L02 cells were cultured in DMEM (Hyclone Laboratories) supplemented with 10% fetal bovine serum (Hyclone Laboratories) at 37°C in an incubator with 5% CO_2_. PDK1 inhibitor GSK-470 and mTORC1/C2 inhibitor PP242 were obtained from Selleck Chemicals (Houston, TX, USA), and dissolved in dimethylsulfoxide (DMSO) at a stock concentration of 10 mg/ml. IGF-1 was purchased from Peprotech (Rocky Hill, NJ, USA).

### PTEN overexpression by recombinant adenovirus

The recombinant adenovirus vectors carrying PTEN gene (Genebank NO: NM_000314) and GFP gene were purchased from Hanheng Biotech (Shanghai, China). RPMI 8226 cells (2×10^5^ cells/well) were seeded in a 6-well plate and transfected with recombinant adenovirus (25 and 50 virus particles/cell). Virus containing solutions were removed after 2 h and the cells were incubated in fresh nutrient solution. Protein expression of PTEN was determined by Western blotting analysis.

### PTEN knockdown by short hairpin RNA

Three recombinant adenovirus vectors containing shRNA against PTEN and a negative control scramble shRNA were purchased from Hanheng Biotech (Shanghai, China). ARP-1 cells (2×10^5^ cells/well) were seeded in a 6-well plate and transfected with adenovirus at a concentration of 100 virus particles/cell. Virus containing solutions were removed after 2 h, and then the cells were incubated in fresh nutrient solution. The sequence of shRNA targeting PTEN was 5′- CTAGAACTTATCAAACCCTTT-3′.

### MTT colorimetric survival assay

Cell viability was determined by 3-(4,5-dimethylthiazol-2-yl)-2,5-diphenyl tetrazolium bromide (Sigma, St. Louis, MO, USA) assay as previously reported [49]. Drug concentrations required to inhibit 50% of cell growth (IC50) was calculated using nonlinear regression analysis.

### RT-PCR assay

Total RNA was extracted using an RNeasy Plus kit (TaKaRa Shuzo, Kyoto, Japan). cDNA templates were made from total RNA using reverse transcriptase kit according to manufacturer's instructions (Invitrogen, Carlsbad, CA, USA). The following specific primer was used to amplify PTEN: Forward, 5′-GACAGCCATCATCAAAGAGATCG-3′; Backward, 5′-CATGGTGTTTTATCCCTCTTG-3′. The PCR reaction was performed using Taq polymerase (Promega) for 35 cycles. PCR products staining by ethidium bromide were fractionated on a 1.5% agarose gel.

### Detection of apoptosis

Cells (2×10^5^) were seeded and incubated overnight at 37°C, 5% CO_2_. Cells were centrifuged at 300*g* for 5 min upon treatment with each drug. Cell pellets were collected and fixed with 70% ethanol on ice for 20 min, followed by centrifugation. Apoptosis were quantified by staining cells with annexin V-FITC and propidium iodide (PI) using annexin V-FITC apoptosis detection kit (BD Pharmingen, San Diego, CA, USA). The samples were analyzed by flow cytometry (FACSCalibur, BD).

### Western blotting

Protein extraction, sodium dodecyl sulfate-polyacrylamide gel electrophoresis, and immunoblotting were performed as described previously [50]. The primary antibodies used were as follows: PDK1, p-PDK1 (Ser241), PTEN, mTOR, p-mTOR (Ser2448), p-mTOR (Ser2481), AKT, p-AKT (Ser473), p-AKT (Thr308), P70S6K, p-P70S6K (Thr389), 4E-BP1, p-4E-BP1 (Thr37/46), PI3Kp110α, poly(adenosine diphosphate-ribose) polymerase (PARP) and Caspase -8, -9, and -3, were purchased from Cell Signaling Technology (Beverly, MA,USA). Monoclonal anti β-actin antibody was purchased from Santa Cruz Biotechnology (Santa Cruz, CA, USA).

### Animal studies

All animal experiments were reviewed and approved by the Institutional Animal Care and Use Committee. RPMI8226 cells (5×10^6^ cells/μl per site) in their logarithmic growth phase were implanted subcutaneously into the right flank of 3-4 weeks-old female severe combined immunodeficient (SCID) mice (Shanghai Experimental Animal Center ofthe Chinese Academy of Sciences, Shanghai, China). Tumor volume was measured and calculated as a previous report. When tumors reached a volume of 80-100 mm^3^, mice were randomly assigned to one of the treatment groups: 5 days of GSK-470 (40 mg/kg/d), 5 days of PP242 (20 mg/kg/d), or 5 days of GSK-470 combined with PP242 as an intraperitoneal injection once every day. The untreated control group received DMSO. After 5 days of treatment, one mouse of each groups were sacrificed, and tumors were harvested for immunohistochemistry.

### Immunohistochemistry

Tumors were fixed in 4% paraformaldehyde, embedded in paraffin, and then cut in 4-mm sections. Detection of p-mTOR (Ser2448) and p-mTOR (Ser2481) in primary tumor samples was performed using the corresponding specific antibodies (Abcam, Cambridge, UK). Apoptotic cells in tumor samples were assessed by TUNEL staining with an In Situ Cell Death Detection kit (Roche, Nutley, NJ, USA) according to the manufacturer's protocol. All tissue sections were counterstained with hematoxylin.

### Statistical analysis

The CI analysis was based on the methods of Chou and colleagues [51]. The synergy of GSK-470 with PP242 was analyzed with the use of CalcuSyn software (Biosoft, Cambridge, UK). The values of tumor volume were expressed as mean ± SD. The difference between groups was analyzed by ANOVA and Student's t-test. *P* < 0.05 was considered to be significant.

## SUPPLEMENTARY FIGURES


