# Supplementary material for: PDK1 inhibitor GSK2334470 exerts antitumor activity in multiple myeloma and forms a novel multitargeted combination with dual mTORC1/C2 inhibitor PP242
Source: Oncotarget. 2017 Mar 29;8(24):39185–97. doi: 10.18632/oncotarget.16642 (PMC5503605; doi:10.18632/oncotarget.16642)
Supplement: Supplementary file 1 [file oncotarget-08-39185-s001.pdf]

PDK1 inhibitor GSK2334470 exerts antitumor activity in multiple myeloma and forms a novel multitargeted combination with dual mTORC1/C2 inhibitor PP242

SUPPLEMENTARY MATERIALS

SUPPLEMENTARY FIGURES

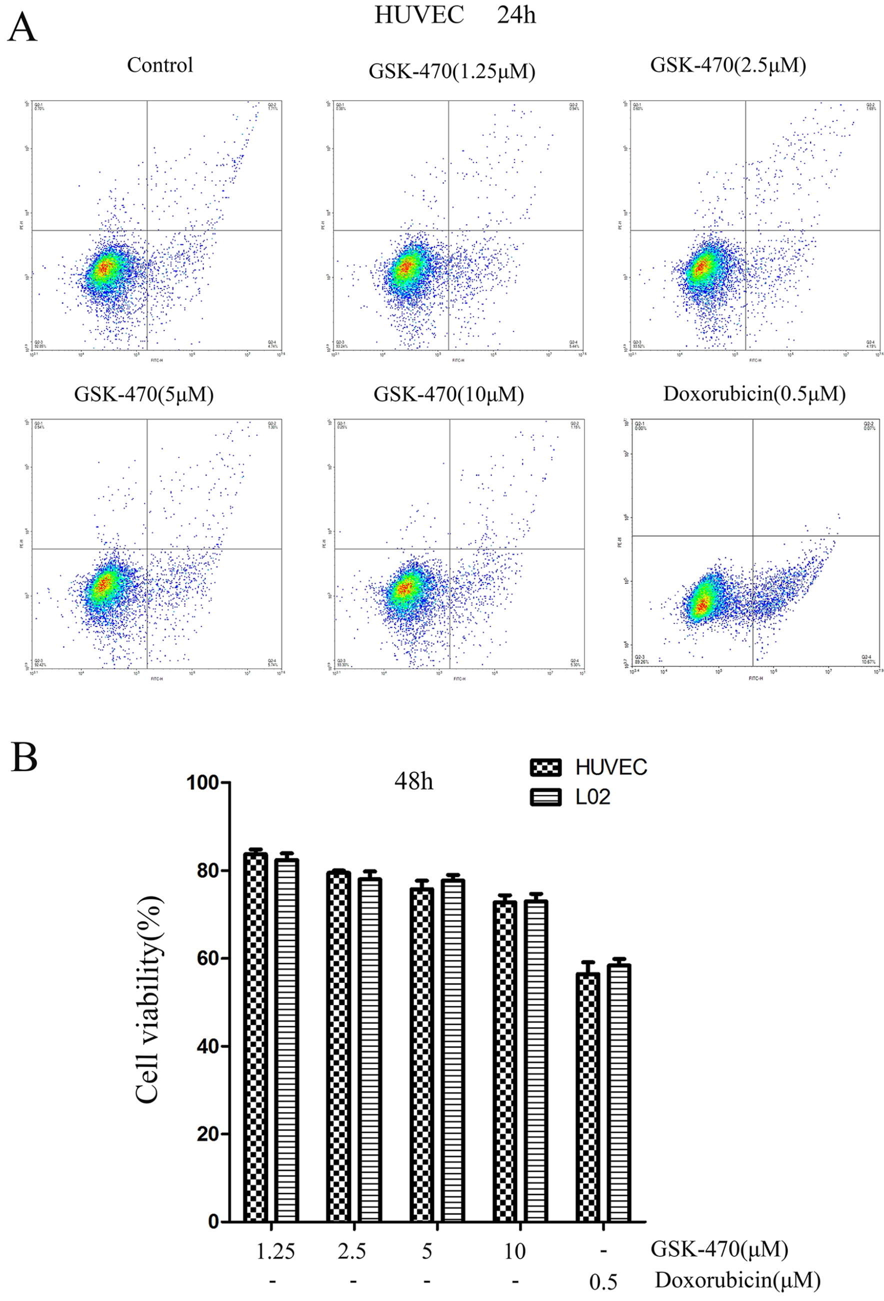

Supplementary Figure 1: The cytotoxicity of GSK-470 on human normal cell lines. (A). HUVEC cells were cultured with different concentrations of GSK-470 for 24 h. Apoptosis was analyzed by flow cytometry after dual staining of cells with annexin V and propidium iodide (PI). **(B)**. HUVEC and L02 cells were cultured with different concentrations of GSK-470 for 48 h. The viability of cells was assessed by an MTT assay. Data are presented as the mean ± SD of three independent experiments.

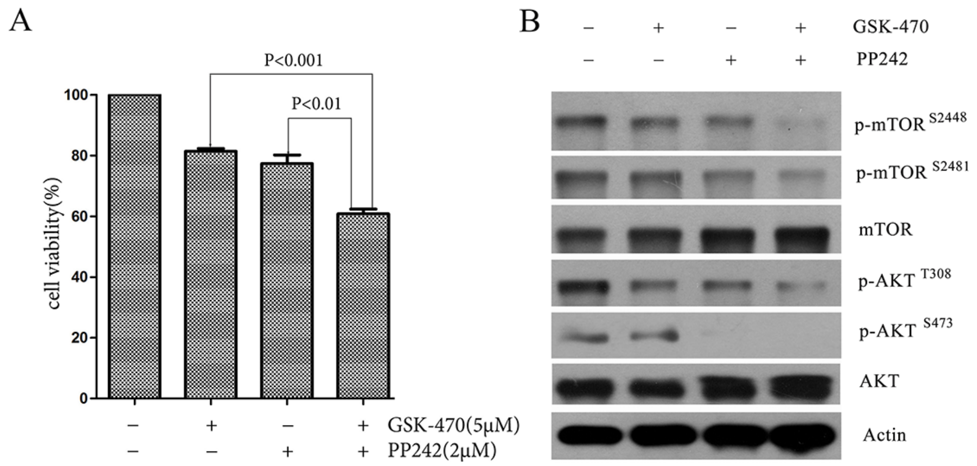

Supplementary Figure 2: The antitumor effect of GSK-470 alone or combined with PP242 on primary myeloma cells. (A). Primary cells were obtained from a 65-year-old patient with newly diagnosed myeloma who has 81% of abnormal plasma cells in bone marrow. The primary myeloma cells were treated with GSK-470 (5 μM), PP242 (2 μM) or in combination for 16 h. The MTT assay was used to determine the viability of cells. Data are presented as the means ± SD of three independent experiments. **(B)**. The expression and phosphorylation of AKT and mTOR were assessed by Western blotting analysis. Actin was used as a protein loading control.
